# Chemoradiotherapy followed by continued immunotherapy for out-of-field progression during consolidation in limited-stage SCLC: a case report

**DOI:** 10.3389/fimmu.2025.1615915

**Published:** 2025-07-10

**Authors:** Ying Jiang, Xu Tong, Nan Bi

**Affiliations:** Department of Radiation Oncology, National Cancer Center/National Clinical Research Center for Cancer/Cancer Hospital, Chinese Academy of Medical Sciences and Peking Union Medical College, Beijing, China

**Keywords:** LS-SCLC, chemoradiotherapy, immunotherapy, out-of-field, progression

## Abstract

**Background:**

While consolidation immunotherapy after chemoradiotherapy (CRT) improves survival in limited-stage small cell lung cancer (LS-SCLC), some patients develop out-of-field progression during consolidation. Optimal management in such cases remains undefined.

**Case presentation:**

We describe a 49-year-old male with LS-SCLC who developed supraclavicular lymph node metastasis during consolidation immunotherapy following definitive CRT. The patient underwent concurrent chemoradiotherapy targeting the recurrent node, followed by continued consolidative immunotherapy. This approach led to complete response of both the primary tumor and the metastatic lymph node, with minimal toxicity.

**Conclusion:**

This case highlights the potential role of combining salvage radiotherapy with continued immunotherapy in managing isolated nodal recurrence during consolidation. Further studies are warranted to validate this individualized strategy.

## Introduction

Immunotherapy consolidation following chemoradiotherapy (CRT) has significantly improved overall survival (OS) and progression-free survival (PFS) in patients with limited-stage small cell lung cancer (LS-SCLC), as demonstrated by the ADRIATIC study (1). Although consolidative immunotherapy has significantly improved PFS, with a hazard ratio for progression or death of 0.76 (P = 0.02), the median PFS remains 16.6 months. Specific data on recurrence patterns were not disclosed, but in clinical practice, some patients have been observed to experience out-of-field recurrences in mediastinal or supraclavicular lymph nodes. According to the National Comprehensive Cancer Network (NCCN) Guidelines for SCLC, for patients with relapsed or primary progressive disease after first-line treatment, systemic therapy or palliative care, including radiotherapy to symptomatic sites, is typically recommended (2). However, the optimal treatment strategy for patients with isolated nodal recurrence during consolidation, including the potential benefit of re-irradiation combined with chemotherapy and immunotherapy, remains unclear. Herein, we present a case of LS-SCLC with supraclavicular lymph node metastasis occurring during immunotherapy consolidation following CRT. The patient subsequently completed localized concurrent CRT targeting the metastatic lymph node, followed by continued consolidative immunotherapy, offering insight into a potential management approach for similar cases.

## Case presentation

The patient was a 49-year-old Asian male with a smoking and drinking history of over 20 years (ceased both after diagnosis). His medical history included fatty liver, liver cysts, and chronic pharyngitis. He was assessed to have an Eastern Cooperative Oncology Group (ECOG) performance status of 0 at the time of diagnosis. In March 2023, he presented with chest tightness, shortness of breath, and a dry cough without other accompanying symptoms. A chest CT revealed a 3.1×2.4 cm nodule in the posterior segment of the right lower lobe, with multiple enlarged lymph nodes in the right hilar and mediastinal regions.

### Clinical findings and diagnostic assessment

Bronchoscopy revealed no visible intraluminal lesions. Endobronchial ultrasound-guided transbronchial needle aspiration (EBUS-TBNA) demonstrated enlarged lymph nodes in the right hilar and mediastinal regions (stations 7, 4R, and 2R). Pathological diagnosis confirmed metastatic small cell lung cancer, with reactive changes observed in station 7 lymph nodes. PD-L1 expression was evaluated as CPS: 1. PET-CT further confirmed the primary tumor and nodal metastases without evidence of distant metastases. Brain MRI was unremarkable. Following evaluation by a multidisciplinary team (MDT) comprising specialists in oncology, thoracic surgery, radiation oncology, radiology, and pathology, the patient was staged as T2aN2M0 and classified as LS-SCLC per the Veterans Administration staging system. The patient was enrolled in a single-arm, open-label, multi-center phase II clinical trial (NCT05443646 (3)) investigating concurrent CRT followed by serplulimab in patients with LS-SCLC.

### Timeline

**Figure 1:**
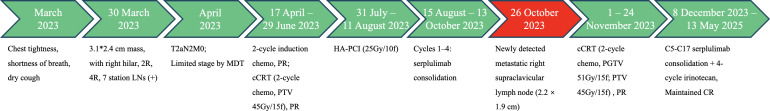
Timeline of the clinical events and treatment strategies. Abbreviation: cCRT, concurrent chemoradiotherapy; Chemo, chemotherapy; LN(s), lymph node(s); MDT, multidisciplinary team; HA-PCI, hippocampal-avoidance prophylactic cranial irradiation; PR, partial response; CR, complete response.

A detailed timeline of the diagnostic, therapeutic, and follow-up processes is illustrated in **Figure 1**.

### Initial treatment

After receiving two cycles of induction chemotherapy with etoposide and cisplatin (EP regimen), the patient proceeded to concurrent CRT with two cycles of EP regimen. Thoracic radiotherapy targeted the primary tumor and mediastinal lymph nodes, delivering a dose of 45 Gy in 15 fractions to the planning target volume (PTV). The gross tumor volume (GTV) was delineated using 4D-CT in combination with pre-chemotherapy PET-CT. Gross tumor volume of nodal (GTVnd) included metastatic lymph nodes in the right hilar, 4R, and 7 stations. The clinical target volume (CTV) was generated by expanding the GTV and GTVnd by 0.5 cm in all directions, covering the right hilar region, mediastinal stations 2R, 4R, and 7, and the corresponding lymphatic drainage areas. The PTV was generated by expanding the CTV by 0.5 cm in all directions. Subsequently, the patient received hippocampal-avoidance prophylactic cranial irradiation (HA-PCI) at a dose of 25 Gy in 10 fractions. Chemotherapy was administered according to NCCN guideline-recommended dosing, and radiotherapy was delivered using volumetric modulated arc therapy (VMAT). Post-treatment imaging indicated partial response (PR) based on RECIST 1.1 criteria. Consolidation immunotherapy with serplulimab was subsequently initiated.

### Recurrence and subsequent treatment

After receiving four cycles of consolidation immunotherapy with serplulimab, follow-up imaging in October 2023 revealed supraclavicular lymph node metastasis. After MDT evaluation, the patient underwent definitive concurrent CRT with two cycles of carboplatin and etoposide concurrently. The C2-GTVnd included the metastatic right supraclavicular lymph node identified on imaging. The planning gross tumor volume (C2-PGTV) was generated by applying a 3-mm isotropic expansion to the C2-GTVnd. The C2-CTV included the C2-GTVnd with a 3-mm margin as well as the regional supraclavicular nodal drainage area. The C2-PTV was created by expanding the C2-CTV uniformly by 3 mm. The prescribed dose was 51 Gy in 15 fractions to the C2-PGTV and 45 Gy in 15 fractions to the C2-PTV. Following the completion of CRT, consolidation immunotherapy with serplulimab was resumed. Additionally, four cycles of irinotecan were administered during the first four cycles of resumed immunotherapy. Post-treatment evaluation demonstrated a complete response (CR) according to RECIST 1.1 criteria.

### Follow-up and outcomes

**Figure 2:**
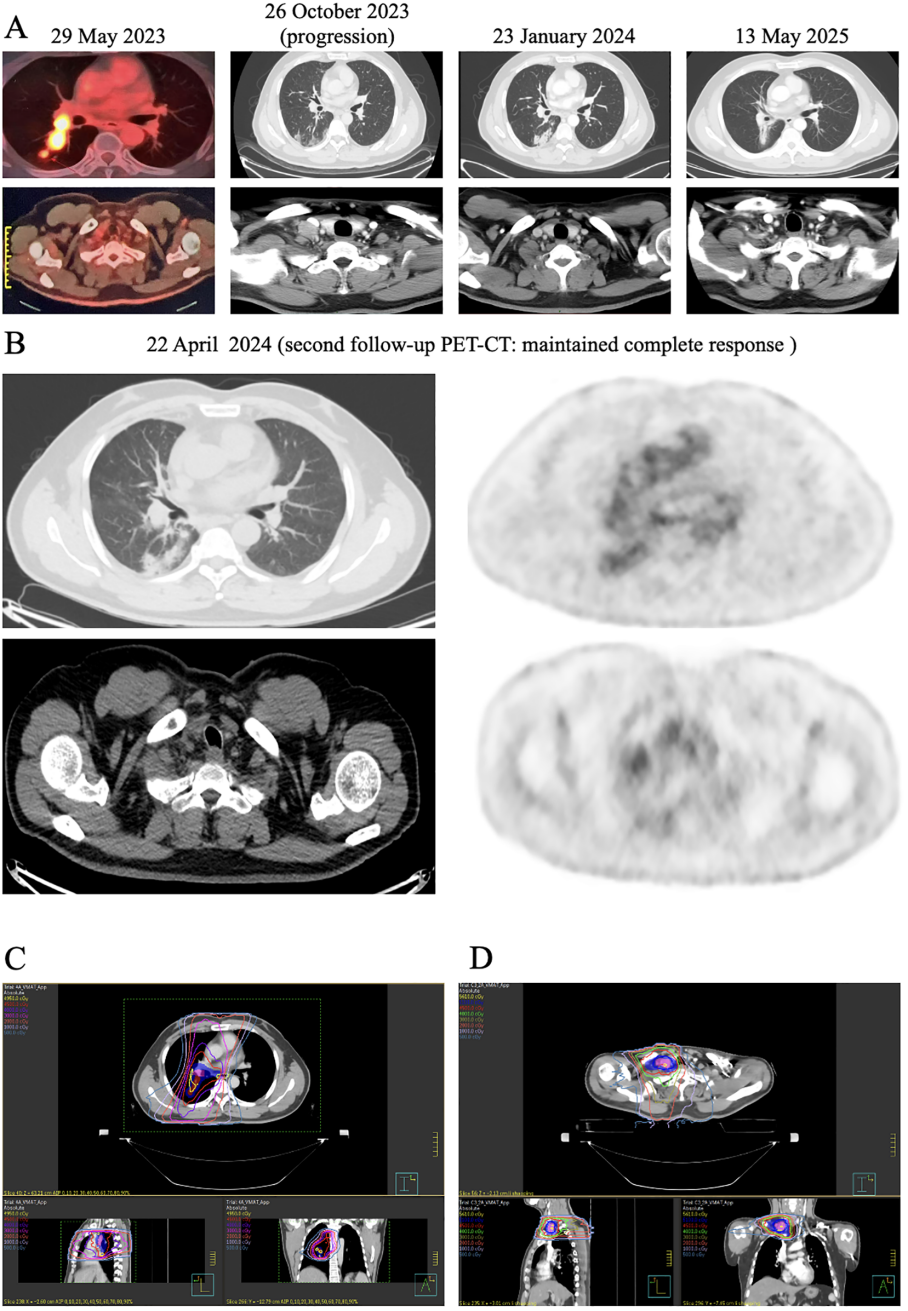
Imaging and RT plans of the patient. **(A)** PET-CT and CT images of the patient at four different time points; **(B)** PET-CT scan of the patient taken on April 22, 2024; **(C)** Chest RT plan; **(D)** Supraclavicular lymph node RT plan. Abbreviation: CT, computed tomography; PET-CT, positron emission tomography-computed tomography; RT, radiotherapy.

After completing 17 cycles of immunotherapy, the patient achieved CR, with complete resolution of the primary lesion and reduction of the supraclavicular lymph node to 0.4 cm (**Figure 2**). As of the most recent follow-up on May 13, 2025, the patient remained in complete response, with no radiological evidence of recurrence or metastasis on contrast-enhanced MRI of the brain and contrast-enhanced CT of the neck, chest, and abdomen. Adverse events included a single episode of grade III ALT elevation, which resolved with symptomatic treatment, and no other significant toxicities.

## Discussion

Despite the encouraging results of the ADRIATIC study (1), some patients with LS-SCLC experience out-of-field progression, such as metastases to mediastinal or supraclavicular lymph nodes. These occurrences pose significant challenges, as the optimal management strategies remain undefined. While systemic therapies like chemotherapy or immunotherapy alone are often considered, this case highlights the potential benefits of a more aggressive approach involving localized definitive chemoradiotherapy targeting the metastatic lesion, combined with continued consolidative immunotherapy. This strategy achieved favorable outcomes in our patient, with both the primary tumor and the metastatic lymph node showing complete response and a tolerable safety profile.

Effective control of the primary tumor suggests that prior CRT and immunotherapy were not ineffective. Therefore, recurrence in out-of-field lymph nodes should not be immediately interpreted as a failure of CRT. Instead, addressing the metastatic lesion with localized interventions may provide the patient with an opportunity for prolonged disease control. Local radiotherapy combined with systemic therapy may offer clinical benefit in selected cases of LS-SCLC recurrence, further research with larger cohorts is needed to confirm these findings.

To our knowledge, there are currently no published case reports describing salvage chemoradiotherapy combined with continued immunotherapy for isolated out-of-field nodal recurrence during consolidation in LS-SCLC. Although some studies in extensive-stage SCLC have explored the use of immune checkpoint inhibitors beyond progression, these findings have not been extended to limited-stage disease (4, 5). This approach warrants further investigation to determine its potential role in the management of LS-SCLC.

Our study has several limitations. First, the ADRIATIC study used the anti-PD-L1 antibody durvalumab as the consolidation drug, whereas serplulimab (HLX10) used in this case is a PD-1 antibody. While serplulimab is currently used as first-line treatment for extensive-stage small cell lung cancer (ES-SCLC) (6), it is still under clinical investigation for LS-SCLC. Second, the follow-up period is relatively short. Longer follow-up is necessary to evaluate the durability of the response.

In conclusion, this case highlights the potential of combining localized CRT with continued immunotherapy to manage out-of-field lymph node progression in LS-SCLC. This individualized approach preserved the opportunity for durable disease control with minimal toxicity. Future studies are warranted to explore the role of localized interventions in similar clinical scenarios and to establish optimized treatment paradigms for LS-SCLC patients with isolated lymph node recurrence.

## Data Availability

The raw data supporting the conclusions of this article will be made available by the authors, without undue reservation.
